# Evidence‐based evolution of an integrated nutrition‐focused agriculture approach to address the underlying determinants of stunting

**DOI:** 10.1111/mcn.12260

**Published:** 2016-05-17

**Authors:** Nancy J. Haselow, Ame Stormer, Alissa Pries

**Affiliations:** ^1^ Helen Keller International Asia Pacific Regional Office Phnom Penh Cambodia

**Keywords:** stunting, nutrition‐sensitive agriculture, homestead food production

## Abstract

Despite progress in reducing hunger and malnutrition since the 1990s, many still suffer from undernutrition and food insecurity, particularly women and young children, resulting in preterm birth, low birthweight and stunting, among other conditions. Helen Keller International (HKI) has addressed malnutrition and household food insecurity through implementation of an Enhanced Homestead Food Production (EHFP) programme that increases year‐round availability and intake of diverse micronutrient‐rich foods and promotes optimal nutrition and hygiene practices among poor households. This paper reviews the evolution and impact of HKI's EHFP programme and identifies core components of the model that address the underlying determinants of stunting. To date, evaluations of EHFP have shown impact on food production, consumption by women and children and household food security. Sale of surplus produce has increased household income, and the use of a transformative gender approach has empowered women. EHFP has also realized nutrition improvements in many project sites. Results from a randomized control trial (RCT) in Baitadi district, Nepal showed a significant improvement in a range of practices known to impact child growth, although no impact on stunting. Additional non‐RCT evaluations in Kailali district of Nepal, demonstrated a 10.5% reduction in stunting and in the Chittagong Hill Tracts in Bangladesh, revealed an 18% decrease in stunting. Based on evidence, the EHFP has evolved into an integrated package that includes agriculture, nutrition, water/hygiene/sanitation, linkages to health care, women's empowerment, income generation and advocacy. Closing the stunting gap requires long‐term exposure to targeted multi‐sectoral solutions and rigorous evaluation to optimize impact.

**Key messages**

- Integrated nutrition‐sensitive and nutrition‐specific programmes, such as HKI's Enhanced Homestead Food Production, can impact stunting by addressing its underlying determinants, including: supporting optimal nutrition, care and health practices among disadvantaged women of reproductive age and infants and young children, reducing food insecurity, empowering women and improving water, sanitation and hygiene.
- Closing the stunting gap requires a mix of interventions based on evidence‐based solutions. Long‐term exposure to these targeted multi‐sectoral solutions and rigorous evaluation is necessary to measure impact on women and young children.

## Introduction

Despite progress in reducing global hunger and malnutrition over the past 25 years, factors such as food price increases, sporadic social and political unrest and increasing inequity have resulted in a stagnation of this progress with many people still suffering from hunger, malnutrition and household food insecurity. According to the Food and Agriculture Organization (FAO), 842 million people in the world still do not have enough to eat, with most of those living in Asia. Asia has the largest number of hungry people – over 550 million (The State of Food Insecurity in the World 2014).

Women and young children remain the most vulnerable to hunger and food insecurity. Undernutrition is responsible for 45% of all under five child deaths or 2.6 million child deaths each year (WHO 2014; Black *et al*. 2013). Hidden hunger, including iron, vitamin A and zinc deficiencies is also still highly prevalent in Asia and has long‐term developmental consequences contributing to the burden of child mortality, poor birth outcomes and lower productivity in affected populations.

According to a recent estimates of child malnutrition, 50 million children under 5 years of age are wasted (too thin for their height) and 159 million children under five (24%) are stunted (too short for their age) in the world today (UNICEF *et al*. 2015). Although global prevalence of stunting decreased from 33% in 2000, the numbers remain alarmingly high. The largest percentage (56%) and number of stunted children (96 million) live in Asia (UNICEF *et al*. 2012).

Stunting can begin before birth. Because most of the growth faltering occurs well before the age of 2 years, it is imperative for programmes to focus interventions that address the causes of child growth during the first 1000 days – from conception through age two – in order to have an impact on stunting. Stunted children become stunted adults who will never realize their full developmental potential. Severe stunting is associated with an IQ loss of 5–10 points (Strauss & Thomas 1998). Low birth weight babies have been found to have IQs five points lower than non‐low birth weight babies (UNICEF 1998).

The Framework for action to achieve optimal fetal and child nutrition and development illustrated in the 2013 Lancet series, identifies household food security (including availability, economic access and use of food), feeding and caregiving resources and practices (including maternal, household and community levels) and access to and use of health services as well as a safe and hygienic environment (i.e. food, care and health) as key determinants of optimal child nutrition, growth and development (Black *et al*. 2013). The Lancet Series also proposed 10 interventions that if implemented at 90% coverage could prevent one million under five child deaths per year, but would avert only 20% of global stunting (Bhutta *et al*. 2013; Ruel *et al*. 2013). Clearly, additional evidence is needed to design an integrated package to achieve a more significant impact on reducing stunting. The interventions found in Box 1 have been categorized as nutrition specific, with direct pathways to nutrition outcomes, and nutrition sensitive, with indirect pathways to nutrition outcomes.

**Box 1. Interventions**

Nutrition Specific

- **Promote appropriate breastfeeding and complementary feeding**
- **Micronutrient supplementation**
- Management of acute malnutrition (including screening and referral)
- Balanced energy and protein supplements to women

Nutrition Sensitive

- **Agriculture & food security**
- Social safety nets
- **Women's empowerment**
- **Water, sanitation & hygiene**
- **Health and family planning services**
- Early child development & child protection programmes

## Evolution of an integrated programme model that links agriculture to nutrition for food security and nutrition outcomes

Over a 25‐year period, Helen Keller International (HKI) has developed an integrated nutrition‐specific and nutrition‐sensitive programme model that uses agriculture as the delivery platform through which disadvantaged households receive a package of services to improve nutritional status among the most vulnerable – young children and pregnant and lactating women. The programme, called Enhanced Homestead Food Production (EHFP), is designed for implementation in rural food insecure areas. It now includes agriculture, nutrition, health, gender and income generation strategies that simultaneously promote optimal nutrition, care and health practices and establishes a system for year‐round food availability and intake of diverse micronutrient rich foods. The model marries production of plant and animal source foods (through home gardens, small animal rearing and agriculture support mechanisms) with nutrition education and behaviour change communication using the essential nutrition actions (ENA) framework, highlighting promotion of optimal breastfeeding and complementary feeding (Guyon *et al*. 2009; WHO 2013). Ensuring that adequate water, hygiene and sanitation interventions are promoted, including uptake of essential hygiene actions (EHA), is also key to optimize the programme's success. The EHFP model targets women, especially pregnant women and those with children under 2 years of age, and empowers them as the primary beneficiaries of training, services and inputs. The model establishes Village Model Farms (VMF) by providing additional training and inputs so the VMF can serve as a sustainable support mechanism for the 10–20 household farmers in her group and become a small private enterprise selling future inputs to the community. A monitoring (multi‐stage cluster random selection of households every 3–4 months) and evaluation system that collects key process and impact data for continual adjustment and programme improvement is integral to the design as well. The EHFP model has evolved over more than 25 years in response to evidence collected by HKI and others and now addresses six of the 10 Lancet Series nutrition‐specific and nutrition‐sensitive interventions (in bold in Box 1), with mechanisms in place for identification and referral of acute malnutrition cases as well.

### Developing the basic homestead food production programme model

The programme was initiated in 1988 as a pilot *home gardening project* in Bangladesh to improve year‐round production and consumption of vitamin‐A rich fruits and vegetables among children under 5 years of age after a national blindness survey revealed that households with gardens were less likely to have children with night blindness (HKI & Institute for Public Health and Nutrition 1983). Based on the pilot evaluation results, the project model was fine‐tuned to include improved gardening techniques for year‐round production and nutrition education to ensure optimal consumption for women and young children, and expanded to additional households. A major improvement to the programme model occurred during the late 1990s, when evidence revealed that the bioavailability of vitamin A and other micronutrients from plant source foods was lower than originally thought – as conversion factors for estimating vitamin A obtained from plant foods were revised from 6:1 to 21:1 for a mixed diet (12:1 for fruits and 26:1 for vegetables) (µg β‐carotene: retinol activity equivalent) (West *et al*. 2002; de Pee & Bloem 2007). In response, the model was revised to include small animal husbandry – mostly poultry, together with promotion of meat and egg consumption among women and young children and named the *Homestead Food Production* (HFP) programme. Breastfeeding and appropriate complementary feeding counselling (using HFP produce) were also incorporated in order to target infant and young child feeding practices. Later, in response to the Avian Influenza outbreak, diversified sources of animal protein were encouraged by testing an aquaculture option in Cambodia and Indonesia. Participants in Bangladesh could also opt to rear pigs or goats in addition to chickens. During these years observational, monitoring and evaluation results from various projects in Asia also informed the programme process and implementation procedures. For instance, the sequence of activities, the duration of exposure to training, technical support and supervision, the kind and amount of agriculture inputs and the key communication materials and messages were all refined.

### Enhancing the nutrition‐sensitive component

In 2007, based on the demonstrated impact of the ENA approach to improve delivery of nutrition‐specific interventions to mothers and young children, HKI revised the HFP model to incorporate ENA as the basic framework for nutrition improvement and renamed it the EHFP programme (USAID 2006; Guyon *et al*. 2009). The new approach included using behaviour change communications approaches to promote key doable actions at every opportunity to enhance uptake: maternal nutrition; early initiation of breastfeeding; exclusive breastfeeding for 6 months; the introduction of adequate complementary foods at 6 months, including the animal‐source foods that are increasingly being shown as crucial for child growth; continued breastfeeding through 2 years or beyond; nutritional care for the sick child; the integrated control of anaemia and intake of key micronutrients (Dewey & Adu‐Afarwuah 2008). Capacity building of and stronger links to the government health sector at health facilities and in communities was undertaken in order to ensure consistency and accuracy of key nutrition messages and to ensure adequate support for health services, better care and referrals particularly for pregnant women and malnourished and sick children.

### Improving household income generation from HFP

Based on a need expressed (during monitoring and supervision visits) by participating women who found that they had remaining produce after household use, HKI added a small marketing component to create linkages and provide training for the sale of excess produce and animal products. This was undertaken with the understanding that even small income in the hands of women can benefits maternal and child nutrition and health (Engle 1993; Roushdy 2004; Quisumbing & Maluccio 2000). Programme monitoring and evaluation (pre and post cross‐sectional surveys comparing intervention and control group) data (Table 1) have shown that this income is in fact often used to purchase high quality and other needed food items or to pay for health and education costs (Talukder *et al*. 2010). All EHFP programmes now include marketing education and training for participants.

**Table 1:** Proportion of households in Bangladesh and Cambodia that spent income earned by selling garden produce, poultry and egg on various items at endline

Household commodities	Bangladesh* (in last 2 months)		Cambodia (in last 1 month)	
	% household spending income from selling home garden products on:	% household spending income from selling egg and poultry on:	% household spending income from selling home garden products on:	% household spending income from selling egg and poultry on:
Food	70	46	92	82
Education	30	26	1	3
Productive/reinvestment	22	25	1	3
Clothes	14	22	0	3
Saving	11	24	0	0
Medicine	8	0	2	6
Housing	1	3	0	0
Amusement	1	2	0	0
Social activities	0	1	1	2
Other	0	0	3	1

* *In Bangladesh, respondents could choose as many commodities as applied.*

Talukder et al., 2010

### Enriching gender equity within EHFP

Although EHFP has been women‐centred from the beginning, monitoring and evaluation data across projects indicated a need to strengthen the approach to better empower women in decision making, to increase male participation in childcare and other traditional women's tasks and to encourage support for mothers from influential family and community members. The Nurturing Connections© manual was created by HKI (in Bangladesh) to implement a transformative gender approach within EHFP (Kotze *et al*. 2014). The approach consists of a range of participatory activities where all main decision‐makers of the household acquire communication and negotiation skills to discuss and challenge traditional norms that impact nutrition, health and overall well‐being of the households.

### Addressing WASH through EHFP

The inclusion of EHA and a stronger water, sanitation and hygiene (WASH) component was a further refinement to the model after the Nepal *Action Against Malnutrition through Agriculture* (AAMA) research project showed less impact on improving child nutritional status and growth than expected (McNulty *et al*. 2013). In addition to the handwashing and diarrhea prevention that already existed in the model, EHA added the creation of handwashing stations, disposal of feces, de‐worming, water purification and proper food storage to the EHFP.

Most recently, HKI has incorporated activities to address environmental enteropathy based on evidence from a randomized control trial (RCT) in Zimbabwe indicating that environmental enteropathies could impact stunting even in the absence of diarrhea (Humphrey 2014). An earlier study conducted with Zimbabwean toddlers found that the greatest cause of intestinal damage, as measured by *Escherichia* *coli*, was because of ingestion of chicken feces (Ngure *et al*. 2013). HKI therefore tested the use of safe and sanitary play areas in Nepal (as was used in the SHINE study), but found that mothers were resistant to the idea that dirt and chicken feces made children sick so there was little interest in uptake despite intensive behaviour change messaging. Instead more than 60 000 chicken coops have been built and are being utilized in project areas (Save the Children & HKI 2014). Further formative research is needed to determine messaging and strategies to improve use of the play areas.

### Better targeting to optimize impact

The AAMA results, along with the Scaling Up Nutrition Movement and the 2013 Lancet series papers, led HKI to prioritize targeting the programme to households with children under 2 years of age and pregnant women in order to address the growth faltering within the 1000‐day window (Scaling up Nutrition 2012; Ruel *et al*. 2013; Bhutta *et al*. 2013; Adair *et al*. 2013). The programme focused even more attention on nutrition services related to interventions that span this critical window from pregnancy into the first 2 years of life (World Bank 2006).

## The EHFP programme impact pathways

Through identification of gaps and testing of new strategies, the components of EHFP have evolved to more comprehensively address the underlying food, health and care determinants of stunting. The current integrated model is expected to improve maternal and child nutritional outcomes through several pathways, namely: (1) increased access to and consumption of micronutrient‐rich fruits, vegetables and poultry or small animal products, (2) improved breastfeeding and complementary feeding practices, (3) increased use of public health services by mothers and children, (4) increased income through the sale of surplus products from the home gardens and small animal husbandry, and (5) empowered mothers by improving their knowledge and skills and influence over household decision making. The model has added a sixth pathway in order to reduce infections (particularly diarrhea disease and intestinal worms) namely (6) improved water, sanitation and hygiene (WASH) practices.

**Figure 1:**
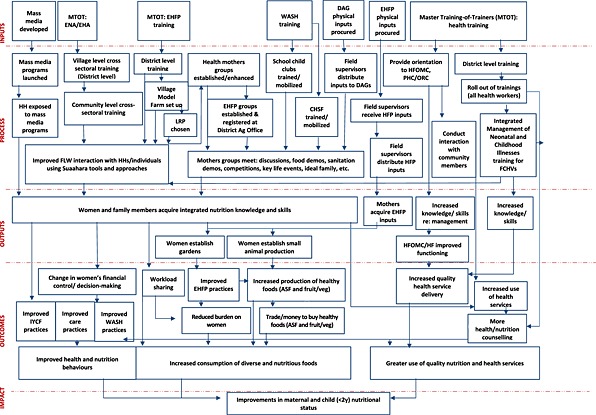
Suaahara Programme Implementation Pathway Helen Keller International, 2012.

The Suaahara project, implemented in disadvantaged areas of Nepal, reflects the current model for HKI's EHFP programme, incorporating critical research findings from AAMA and other research to try to optimize the impact on stunting. The Suaahara project is a 5‐year USAID funded project with Save the Children and other partners and uses the ENA framework to improve nutrition; maternal, newborn and child health (MNCH) services; reproductive health/family planning services; WASH and home‐based food production in 41 districts throughout the country. Figure 1 below describes the Suaahara EHFP Programme Impact Pathways.

## Impact of HKI's EHFP on determinants of stunting

EHFP across the Asia‐Pacific region has improved household garden and animal husbandry practices, food production, consumption and dietary diversity among women and young children. EHFP has also been shown to improve uptake of essential nutrition and hygiene actions by mothers and other caretakers, improve linkages to the health sector and enhance women's decision making power in the household. EHFP has been shown to reduce anaemia and nightblindness (vitamin A deficiency) prevalence among young children and women of reproductive age in some projects and has improved women's nutrition; however, more evidence on its impact on child growth is needed.

**Figure 2:**
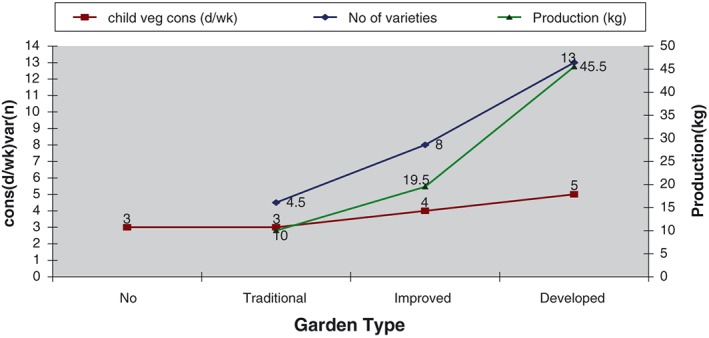
Type of garden^1^ 1Traditional gardens are seasonal in scattered plots and only produce gourd and traditional vegetables. Improved gardens produce a number of vegetables in fixed plots, but not throughout the year. Developed gardens produce a wider range of vegetables in fixed plots throughout the year related to production and consumption of vegetables in Bangladesh (2003–2005) and Cambodia (2005–2007) at endline (consolidated).

A review of EHFP programme evaluations (pre and post cross‐sectional surveys comparing intervention and control group) from 2003 to 2007 in Bangladesh, Nepal, Cambodia, Indonesia and the Philippines indicates that HKI's EHFP has positively impacted poor households' year round food production and availability, particularly for women and children 6–59 months of age (Talukder *et al*. 2010). As illustrated in Fig. 2 below, programme evaluation research by HKI in Bangladesh and Cambodia showed that as gardening practices improved from traditional gardening (seasonal with limited crops grown) to developed gardening promoted by HKI (year round production with a variety of crops grown at all times), the quantity and number of vegetables varieties grown increased, as did consumption of vegetables by children. The diversity of vegetable consumption by young children in the week before the survey was four types eaten when households practiced traditional gardening compared with 13 types eaten when households practiced developed gardening. Frequency of consumption of vegetables by children was 1.6 times higher among children in households with developed gardens relative to traditional gardens. Consumption of vitamin A rich foods among children was also improved (Talukder *et al*. 2010).

The results also demonstrated an increase in women's level of influence in household decision‐making along with increased household income controlled by women (Talukder *et al*. 2010).

**Figure 3:**
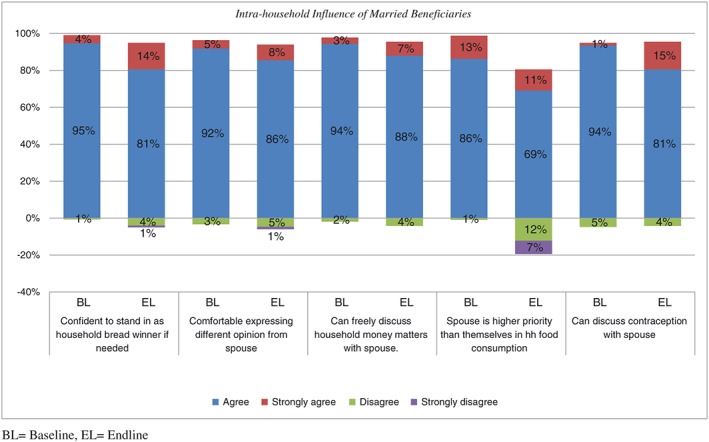
Women's Empowerment Making Markets Work for Women Final Report. 2012.

An external evaluation (comparing current HFP households, former HFP households and controls) in Bangladesh confirmed that through HFP women's influence in the household improved across all parameters as women were empowered with increased knowledge and skills and also were able to control a small amount of income from sale of excess EHFP produce (Bushamuka *et al*. 2005). A more recent evaluation of the pilot project, Making Markets Work for Women (M2W2), an EHFP and livelihood intervention with 450 extremely poor landless women in Bangladesh, confirms these findings (HKI 2012). As seen in Fig. 3 below, endline data revealed that respondents had higher levels of comfort with expressing opinions and discussing money matters and family planning issues than at baseline. Additionally, a recent qualitative assessment has indicated that households participating in Nurturing Connections as part of EHFP share household food more equitably, pregnant and lactating women receive support for domestic duties from other household members, and household members engage more in joint decision‐making regarding food purchases and use of income (HKI‐Bangladesh 2015).

An external review/secondary data analysis of HKI's EHFP in Bangladesh by the International Food Policy Research Institute (IFPRI) revealed that the programme increased diversity and quantities of food produced and consumed, and also increased women's involvement in household decision making, noting, ‘*there is sufficient evidence to conclude that* (EHFP) *is improving household food security, and in some cases nutrition and other intermediary outcomes*’ (Iannotti *et al*. 2010). Intake of micronutrient rich fruits and vegetables by mothers and young children among poor families in Bangladesh improved substantially in just 1 year, as a result of additional production year round and promotion and instruction of how to prepare the foods for children (Talukder *et al*. 2000). Evaluations of other nutrition‐sensitive agriculture interventions support these findings, showing consistent improvements in the volume and diversity of vegetables and fruits produced, diversity of foods consumed and women's involvement in family decision‐making, all of which are determinants for child health (Masset *et al*. 2012).

To address the dearth of evidence on nutrition‐sensitive agriculture interventions impact on nutrition status, HKI designed a study to assess the impact of the EHFP model on child nutrition and growth (anthropometry), as well as women's nutritional status. As part of a USAID‐funded Child Survival Innovations Project, HKI implemented two EHFP studies in Nepal (AAMA Project) from 2009 to 2012 in Baitadi and Kailali districts that addressed the food, health and care causes of malnutrition in the area. The project, covering approximately 11 000 households, was an integrated EHFP model including home gardens, poultry‐rearing, ENA behaviour change communication led by community health workers, a hygiene component and activities to improve linkages to health services.

The main research study within the AAMA project was a cluster RCT that assessed more than 2000 families with children 12–48 months of age at baseline and endline in intervention and control areas. Results showed that there was a significant improvement in household food security (using the Household Food Insecurity Access Scale; Coates *et al*. 2007) among intervention households compared with controls, with the greatest difference realized among severely food insecure households. EHFP improved early initiation of breastfeeding, exclusive breastfeeding, complementary feeding practices, hygiene practices of mothers and children and had a significantly positive impact on children's/mother's participation in preventative health services as well. At endline, anaemia was significantly lower among mothers and children in the intervention (EHFP) group, and mothers participating in the EHFP were significantly less likely to be underweight (BMI < 18.5 kg/m^2^) than those who were not in the programme. However, there was no impact seen on child anthropometry (Haselow & Osei 2014).

The study did show that an integrated agriculture‐nutrition focused intervention can play an important role in improving determinants of stunting such as household food security, maternal hand‐washing and child feeding practices, as well as reducing anaemia among women and children and decreasing maternal underweight.

**Figure 4:**
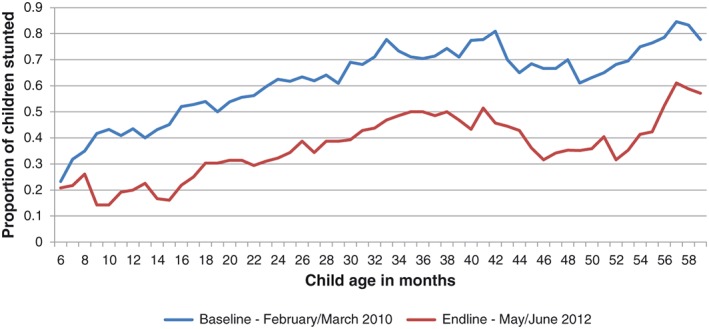
Percentage of stunted children at baseline and endline, Making Markets Work For Women, HKI Bangladesh.

The Kailali district project was not a cluster randomized controlled trial, but did use an intervention–comparison evaluation design. Difference in difference analysis between intervention and control groups showed a reduction of 10.25% (*P* value 0.008) in stunting between baseline and endline. Furthermore, the M2W2 evaluation in Bangladesh demonstrated a reduction in stunting among participating households (see Fig. 4 below). The baseline and endline surveys revealed that 54% of children suffered stunting at baseline and, of those, 19% were severely stunted. By endline stunting had been reduced to 36%, and severe stunting was reduced to 16% (HKI 2013). However, there was no control group for the project and because of the census methodology utilized – some of the same children were measured in households at base and endline, which could lead to ageing effects, distorting the true impact.

Despite these positive results from EHFP, data across nutrition‐sensitive agriculture programmes have shown inconsistent impact on nutritional status of children, and in particular they have shown little impact on child growth. Some reasons for this may be that few evaluations of agriculture programmes have included nutritional indicators, and those that have, have not utilized sufficiently rigorous evaluation designs in order to detect change (Masset *et al*. 2012). In addition, both the duration of the exposure to the intervention before evaluating the impact and targeting of the intervention may have been suboptimal. Programmes may not have addressed all of the aspects included in the three determinants of malnutrition adequately, namely food, care and health (including adequate attention to ensuring clean water and proper sanitation and hygiene) (UNICEF 1997). Finally, the indicators used to measure the determinants may not have been specific enough to capture an association with stunting (i.e. ownership of toilet vs. use of toilet). In the case of the AAMA trial mentioned above, reasons for lack of impact seen on child growth may be that the design was cross‐sectional rather than longitudinal, the age range of children studied may have been too broad and too old and the duration of exposure to the intervention may have been too short to show impact. In addition, the WASH component of the integrated model may not have been adequate to control infection.

## Lessons learned from implementing a nutrition‐focused agriculture programme model

HKI's EHFP programme has been replicated and adapted to the local context to reach over 1.2 million households in Bangladesh, Cambodia, Indonesia, Nepal, the Philippines and Vietnam as well as to three countries in Africa. Adapting and scaling the programme to incorporate evidence that leverages impact on the underlying determinants of stunting have led to the following key learnings summarized in Box 2 and expanded below:

1. Agriculture is critically important but not enough to improve nutrition of vulnerable populations in food insecure areas. For agriculture interventions to optimize nutrition outcomes of the most vulnerable, they should be designed through a nutrition lens, addressing local food, care and health determinants of malnutrition. This requires working with partners across sectors to ensure all aspects of the local determinants of undernutrition are adequately addressed.
2. It is crucial to target pregnant women and women with children under 2 years of age. This targeting focuses attention on the critical 1000‐day developmental window and builds women's knowledge and confidence to make good nutrition and health choices for their children and themselves.
3. Geographic targeting is also important considering the cost of inputs and training required: nutrition focused agriculture programmes have the greatest impact when implemented in food insecure areas among disadvantaged households.
4. In order to maximize health and nutrition outcomes for women and children, programmes should be designed to empower women in their important role as the gatekeepers of household food security, food production, and hygiene and child nutrition. This also means educating and enlisting other influential members of the household, such as mothers‐in‐law and husbands, to provide both emotional and physical support.
5. The design and adaptation of the integrated programme delivery model should be based on evidence from the project area.

a An equity analysis should be performed to provide an understanding of the social/gender dimensions that must be addressed to implement effective programmes that reduce gender/social barriers and enable men and women to better access resources, livelihood opportunities and improved nutrition and food security.
b The behaviour change communication strategy should be based on formative research and tested to facilitate lasting change. The research should include an analysis of the barriers and facilitators for improved nutrition and should be used to develop a strategy for positive change regarding agriculture, nutrition, health, WASH and livelihoods behaviours. The messages should be consistent and reinforced through multiple channels (interpersonal communication, community mobilization, mass media) at all levels.
c A practical training package for agriculture, nutrition and health should be adapted to the local context and tested. The training content should be updated to include state of the art information and appropriate technology in nutrition, health, WASH and agriculture that could be usable and available in the project area. Agriculture practices and planning should address climate change issues in the target area.

d Have an entry and exit strategy. Participatory and learning methods are essential for community entry and to ensure full participation and ownership among stakeholders. There should be an initial and ongoing support mechanism built into the programme model with a qualified technical and management team available to build capacity of local resources in order to ensure households can receive the support they need, and be prepared to continue without much assistance.
e A local source for continued agriculture inputs and support is important for access (particularly in more remote areas), affordability and sustainability of the agriculture component. It is best when these are civic‐minded, community‐selected households that receive additional training and can establish a VMF as a private household enterprise.
f Building multi‐sector collaboration at national, regional and local level is important to maximize nutrition outcomes and use limited resources wisely. Locally, this also means that links to existing agriculture resources, both private and public sector, and to markets must be established in the project. Strong links to the existing health, nutrition and WASH resources should be embedded in the project, as well as working closely with local authorities to support community improvements and leverage additional resources.
g A monitoring and evaluation system that provides continuous learning is necessary when adapting the model successfully and sustainably to new areas, environments and cultures. Nutrition indicators should be included in any intervention with the aim of improving nutrition, including agriculture projects. A rigorous evaluation designed specifically to assess impact on nutrition is important in order to build the evidence base for integrated approaches.
h Documentation, publication and dissemination are critical to advocate for policies that support integrated nutrition‐specific and ‐sensitive approaches and to compel donors to invest in these kinds of interventions for poor communities.

Box 2. Key learning to leverage impact of nutrition‐focused agriculture programmes

1. Collaborate with local partners across sectors to ensure all food, health and care determinants of undernutrition are adequately addressed
2. Collaborate with local partners across sectors to ensure all food, health and care determinants of undernutrition are adequately addressed
3. Target interventions to pregnant women and women with children under two years of age to best address child growth
4. Implement in food insecure areas among disadvantaged households
5. Ensure the programme design empowers women
6. Base the programme design and adaptation to the local context on evidence
7. Have a programmatic entry and exit strategy
8. Ensure there is a local source for continued agriculture inputs and support
9. Work to build multi-sector collaboration at national, regional and local levels
10. Provide continuous learning through an effective monitoring and evaluation system
11. Document, publish and disseminate findings to advocate for policy and resource support

## Toward a more comprehensive strategy to close the stunting gap

In light of the 80% gap that remains in reducing child stunting based on the Lancet series estimation, more work is needed to design an integrated nutrition‐specific and nutrition‐sensitive strategy that intensifies the current known solutions, improves targeting and/or includes additional interventions that may leverage impact on stunting. This will also require that all sectors, public and private, form effective partnerships and do their part to deliver quality services to disadvantaged communities and households.

HKI is continuing to assess and refine the integrated EHFP model to help fill the gap. We are currently undertaking a number of studies to look at various aspects of the model, including several RCTs to assess its impact on child growth. We are also conducting cost analysis of different versions of the model, mapping the pathway by which income contributes to improved nutrition, assessing the spin off effect to demonstrate the potential scale up of the model to neighbouring households, and assessing the sustainability of the model and model components at different times post project.

An important consideration is to begin targeting of interventions to prevent stunting earlier, before pregnancy. Previous research has mostly assessed growth faltering of children after birth, demonstrating that most of the growth faltering occurs once complementary feeding begins. HKI is currently engaged in a study in Bangladesh to enroll women at marriage and assess the impact of EHFP on maternal nutrition and child growth. More research is needed on the impact of stunting beginning at the first day of the 1000‐day period mark, and the impact of reaching adolescent girls and women before they are pregnant.

In many cases, the short length of exposure to nutrition‐sensitive interventions may not be adequate to determine changes in stunting in field conditions. Because of project and research funding cycles, few, if any, evaluations take place for extended periods of time. HKI has recently initiated long‐term projects (more than 5 years) involving rigorous evaluation and surveillance mechanisms in Cambodia and in Bangladesh to look at impact over time.

Another important need is to refine measures that have already been identified in previous studies in the areas of diarrhea, WASH and food consumption so that the correct indicators on compliance and uptake are measured. For instance, measuring the presence of a latrine or availability of soap and water at households is notadequate to assess whether people actually use the latrine or wash hands after defecation and before cooking/eating, which will increase exposure and repeat parasitic infections.

Additionally, the body of evidence should include both monitoring and evaluation data to better understand where issues of uptake and compliance happen so that programme models, such as HKI's EHFP, can be refined to achieve a greater impact. A greater number of comprehensive, rigorous programme evaluations conducted, which are long term and longitudinal, must be designed and funded to ensure that the fight to reduce stunting is based on evidence.

Finally, one of the areas warranting further investigation is the relationship between maternal depression and exposure to stressful events during and post pregnancy as contributing factors to child nutrition and growth. A recent meta‐analysis revealed that maternal depression was linked to child stunting and underweight in developing countries (Surkan *et al*. 2011). Other studies found that stressful events occurring during the first and second trimesters of pregnancy increased risk of preterm birth and perceived stressful events during first trimester were associated with a 99.09‐g decrease in infant birthweight (Glynn *et al*. 2008; Littleton *et al*. 2010; Zhu *et al*. 2010). Exposure to stress and anxiety remains to be evaluated as part of nutrition‐sensitive interventions, and measures to do so will need to be adapted to the developing country context. Additional areas that might contribute to a more effective approach to reduce stunting include strategies to improve resilience specifically of disadvantaged communities and households and better convergence of nutrition‐sensitive interventions by multi‐sectoral stakeholders in these communities.

## Conflicts of interest

The authors declare that they have no conflicts of interest.

## Contributions

NJH drafted the manuscript, AS contributed to the writing and AP provided valuable information for key sections. All authors reviewed, edited and approved the final submission.
